# Multivalent and Sequential Heterologous Spike Protein Vaccinations Effectively Induce Protective Humoral Immunity against SARS-CoV-2 Variants

**DOI:** 10.3390/vaccines12040362

**Published:** 2024-03-27

**Authors:** Rong Liu, Janhavi P. Natekar, Ki-Hye Kim, Heather Pathak, Noopur Bhatnagar, Jannatul Ruhan Raha, Bo Ryoung Park, Anchala Guglani, Chong Hyun Shin, Mukesh Kumar, Sang-Moo Kang

**Affiliations:** 1 Center for Inflammation, Immunity & Infection, Institute for Biomedical Sciences, Georgia State University, Atlanta, GA 30303, USA; rliu12@student.gsu.edu (R.L.); kkim39@gsu.edu (K.-H.K.); nbhatnagar1@gsu.edu (N.B.); jraha1@student.gsu.edu (J.R.R.); bpark9@gsu.edu (B.R.P.); cshin@gsu.edu (C.H.S.); 2 Department of Biology, College of Arts and Sciences, Georgia State University, Atlanta, GA 30303, USA; jnatekar1@student.gsu.edu (J.P.N.); hpathak1@gsu.edu (H.P.);

**Keywords:** SARS-CoV-2, spike, sequential heterologous vaccination, multivalent vaccine, neutralizing activity

## Abstract

The emergence of new SARS-CoV-2 variants continues to cause challenging problems for the effective control of COVID-19. In this study, we tested the hypothesis of whether a strategy of multivalent and sequential heterologous spike protein vaccinations would induce a broader range and higher levels of neutralizing antibodies against SARS-CoV-2 variants and more effective protection than homologous spike protein vaccination in a mouse model. We determined spike-specific IgG, receptor-binding inhibition titers, and protective efficacy in the groups of mice that were vaccinated with multivalent recombinant spike proteins (Wuhan, Delta, Omicron), sequentially with heterologous spike protein variants, or with homologous spike proteins. Trivalent (Wuhan + Delta + Omicron) and sequential heterologous spike protein vaccinations were more effective in inducing serum inhibition activities of receptor binding to spike variants and virus neutralizing antibody titers than homologous spike protein vaccination. The higher efficacy of protection was observed in mice with trivalent and sequential heterologous spike protein vaccination after a challenge with a mouse-adapted SARS-CoV-2 MA10 strain compared to homologous spike protein vaccination. This study provides evidence that a strategy of multivalent and sequential heterologous variant spike vaccination might provide more effective protection against emerging SARS-CoV-2 variants than homologous spike vaccination and significantly alleviate severe inflammation due to COVID-19.

## 1. Introduction

The pandemic outbreak of coronavirus disease 2019 (COVID-19) was caused by severe acute respiratory syndrome coronavirus 2 (SARS-CoV-2). The SARS-CoV-2 spike protein, which is a major target for COVID-19 vaccination, has a receptor-binding domain (RBD) interacting with human angiotensin-converting enzyme 2 (hACE-2) to gain an entry into the host target cells. Initial COVID-19 vaccinations have been successful in providing protective immunity against earlier SARS-CoV-2 pandemic variants (Alpha, Beta, Gamma) with few mutations [1]. SARS-CoV-2 continued to mutate into the Delta variant in mid-2021, and became a dominant strain until the end of 2021. Individuals retaining high titers of neutralization after boost vaccinations, natural infection, or both vaccination and infection could confer protection against severe COVID-19 by these earlier variants, despite-immune escaping mutations and breakthrough infections [2,3]. The ongoing mutation of SARS-CoV-2 resulted in the Omicron variant emerging in November 2021, which established dominance globally soon after [4]. High breakthrough infections with the Omicron lineage variants were reported in vaccinated individuals due to unexpectedly numerous escape mutations [5,6,7].

Approximately 70% of the world population has been vaccinated with at least one dose of the SARS-CoV-2 vaccines as of April 2023 [8]. Despite high coverage rates of vaccinations and multiple shots with COVID-19 vaccines (Pfizer, Moderna, AstraZeneca, Novavax, CoronaVac), the vaccine protective efficacy dropped below 50% upon the emergence of the new Omicron lineage variants [9]. Circulating Omicron variants, including BA.1, BA.2, BA.4/5, XBB.1.5, XBB.1.16, and XBB.1.9.1, are highly immune evasive, leading to breakthrough infections [10,11]. To better protect against the emerging Omicron variants, the FDA has approved former bivalent and monovalent (XBB.1.5) COVID-19 vaccines as a booster dose, which is now in a transition to regular vaccinations. Bivalent booster (Wuhan + BA.1 or BA.4/5 Fall 2022: no longer available) was shown to provide benefit in decreasing COVID-19 hospitalizations. However, the overall relative efficacy of the bivalent booster was not significant in preventing SARS-CoV-2 Omicron infection among persons who were immunized with one or two prior monovalent Wuhan booster vaccinations or recovered from a prior Omicron infection [12].

Therefore, it is critically important to better understand the impact of pre-existing immunity on inducing more broadly neutralizing antibodies by booster vaccination. Also, a more effective vaccination strategy remains to be developed for better control of the ongoing emergence of new Omicron variants. In this study, we tested the hypothesis that vaccination strategies of sequential heterologous boost vaccinations in individuals primed with Wuhan strain or multivalent spike protein vaccinations would induce broader neutralizing antibodies and improve protective efficacy. A strategy of multivalent and sequential heterologous spike protein vaccinations was found to be more effective than homologous spike protein vaccination in inducing protective humoral antibodies of inhibiting hACE2 receptor binding to spike variants, virus neutralization, and protection against SARS-CoV-2 in a mouse model.

## 2. Material and Methods

### 2.1. Recombinant Proteins and Reagents

SARS-CoV-2 variant full length ectodomain spike with pre-fusion stabilizing mutations and receptor binding domain (RBD) proteins were purchased from Sino Biologicals (Wayne, PA, USA): Wuhan (40589-V08B1, S1+S2 ECD, 134.36 kDa, recombinant baculovirus), Delta (40589-V08B16, S1+S2 ECD, 134.20 kDa, recombinant baculovirus); Omicron BA.1 (40589-V08H26, S1+S2 ECD, 136.67 kDa, HEK293 Cells); Omicron BA.2 (S1+S2) (40589-V08H34); Omicron BA.4/5 (S1+S2) (40589-V08H32); Omicron XBB.1.5 (S1+S2) (40589-V08H45); Omicron RBD (40592-V08H121); human angiotensin-converting enzyme 2 (hACE2) protein (aa 1–740) fused to Fc tag (10108-H02H). Full-length ectodomain spike recombinant proteins (Wuhan, Delta, Omicron BA.1) displayed high levels of hACE2 receptor binding activity, with the Wuhan spike protein showing higher activity than Delta and Omicron BA.1 spike proteins, suggesting the integrity of the spike protein folding (Supplementary Figure S1). Wuhan RBD (NR-52366) was obtained from BEI (Biodefense and Emerging Infections Research Resources Repository). Monophosphoryl lipid A (MPL) and saponin QS-21 adjuvants were obtained from Sigma-Aldrich and Desert King (San Diego, CA, USA), respectively.

### 2.2. Vaccination

BALB/c mice (6–8 weeks old female) were purchased from the Jackson Laboratory (Bar Harbor, ME, USA). Animal experiments were performed under the institutional animal care and use committee approved protocol (A23049). Mice were intramuscularly (I.M.) vaccinated with sequential prime–boost–second boost of 1 µg Wuhan (W) and variant spike protein (1 µg): W-Delta (D)-D, W-D-Omicron (O), W-O-O. Trivalent (Tri) groups were prime–boost–second boost vaccinated with combined 1 µg W + 1 µg D + 1 µg O (Tri 1 µg) or 0.33 µg (Tri 0.33 µg) of each W (0.33 µg) + D (0.33 µg) + O (0.33 µg) spike proteins. AS-01 like adjuvant QS21 (10 µg) plus MPL (1 µg) was included in all vaccination and mock control groups. Blood samples were collected 2 weeks after each immunization and stored at −20 Celsius for humoral immune assays.

### 2.3. Inhibition Assay of hACE2 Binding to Spike Proteins 

We carried out the receptor binding inhibition assay to determine the functional antibody levels of vaccine antisera in blocking the interaction between hACE2 and spike proteins, as described previously [13]. Briefly, the recombinant full-length spike protein of Wuhan, Delta, and Omicron variants were coated on a 96-well plate (50 ng/100 uL/well). Vaccine antisera at 3-fold dilutions were added to the spike protein-coated plates and incubated at room temperature for 2 h before incubation with hACE2-Fc (0.5 µg/mL). The hACE2 binding inhibition reactivity was determined by using anti-human IgG conjugated to horse radish peroxidase.

### 2.4. Enzyme-Linked Immunosorbent Assay (ELISA)

IgG antibodies specific for spike full-length protein and RBD domain were determined by ELISA as we described previously [14]. The levels of IgG antibodies were presented in concentrations based on the known IgG antibody controls (Southern Biotech, Birmingham, AL, USA). In brief, purified mouse IgG was used to obtain the standard curve equations of IgG concentrations versus optical density (OD at 450 nm) values. Serum dilutions within a linear correlation range of OD values and IgG levels were applied to calculate IgG concentrations in each sample.

### 2.5. SARS-CoV-2 Challenge 

All the SARS-CoV-2 infection animal experiments were conducted in a certified Animal Biosafety Level 3 (ABSL-3) laboratory at Georgia State University (GSU). Vaccinated and mock control mice (*n* = 5) were intranasally challenged with SARS-CoV-2 (MA10 virus) (1 × 10^5^ PFU, plaque-forming units) at 8 weeks after second boost. Challenged mice were daily monitored for recording weight changes. On day 3 post-challenge, mice were euthanized using isoflurane and perfused with cold PBS. Lung was harvested and frozen in 2-methylbutane (Sigma, St. Louis, MO, USA) for future analysis.

### 2.6. Lung Viral Titration 

Lung viral RNA levels were measured by using qRT-PCR with primers and probes specific for the SARS-CoV-2 N gene, as previously described [15]. Viral genome copies in the lung were analyzed by comparing to a standard curve produced using a known RNA amount extracted from a titrated SARS-CoV-2 sample. Frozen tissues from all groups were weighted and lysed in RLT buffer (Qiagen), and the Qiagen RNeasy Mini kit (Qiagen, Germantown, MD, USA) was used to extract RNA. The whole amount of tissue RNA was quantified and normalized, and the result is shown in viral RNA levels per ug of total RNA.

### 2.7. Plaque Reduction Neutralization Test (PRNT)

SARS-CoV-2 neutralizing antibodies titers in immune sera were investigated using the PRNT assay, as described previously [16]. Immune sera were diluted serially from 1:4 to 1:5120. Wuhan (BEI NR-52281) and Omicron BA.1 strains of SARS-CoV-2 were used to conduct the PRNT assay. To determine the concentration of sera resulting in a 50% plaque reduction, we compared the results with the growth of virus control.

### 2.8. Cytokine and Chemokine Analysis 

Lung tissues were homogenized in a bullet blender (Next Advance, Averill Park, NY, USA), as previously reported [17]. Cytokine and chemokine levels (GM-CSF, IFN-γ, IL-1α, IL-1β, IL-2, IL-4, IL-5, IL-6, IL-7, IL-9, IL-10, IL-12, IL-15, IL-17, IP-10, MCP-1, G-CSF, KC, MIP-1α, MIP-1β, MIP-2, TNF-α, and RANTES) were measured using a MILLIPLEX Mouse Cytokine/Chemokine Magnetic Bead Panel (MilliporeSigma, Temecula, CA, USA). Five-parameter logistic of spline curve-fitting method standard curves were generated, and the samples’ concentrations of cytokines and chemokines were calculated using MILLIPLEX Analyst 5.1 software (Millipore).

### 2.9. Statistical Analysis

Statistical significance for all IgG ELISA, neutralization antibody titers, body weight change, lung viral titration was performed by one- or two-way analysis of variance (ANOVA). GraphPad Prism 9 software (GraphPad Software, Inc., San Diego, CA, USA) was used to calculate the *p* values of the difference between each group. The *p* value is presented by lines (*; *p* < 0.05, **; *p* < 0.01, ***; *p* < 0.001, ****; *p* < 0.0001).

## 3. Result 

### 3.1. Heterologous or Trivalent Spike Protein Vaccinations More Effectively Induce Cross-Reactive IgG Antibodies Than Wuhan Spike Homologous Vaccine

**Figure 1:**
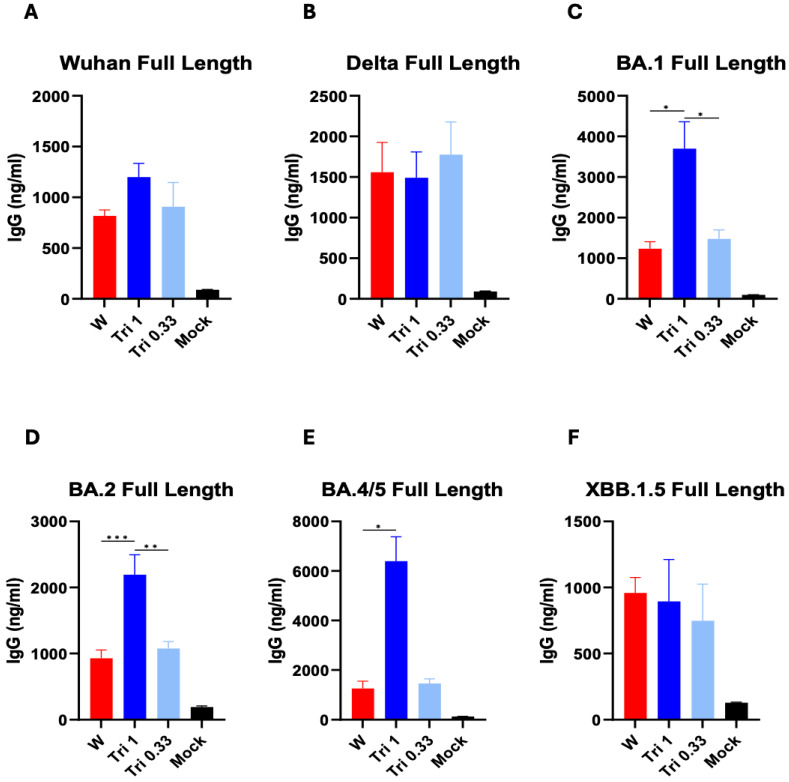
Spike protein-specific IgG antibody responses in immune sera after prime immunization. IgG antibody levels in antisera collected at 2 weeks after prime IM immunization of mice (*n* = 5), as determined by ELISA coated with full-length spike Wuhan (**A**), Delta (**B**), or Omicron variant (**C**–**F**) protein. W: Wuhan (1 µg), Tri 1: W (1 µg) + D (1 µg) + O (1 µg), Tri 0.33: W (0.33 µg) + D (0.33 µg) + O (0.33 µg). Mock: adjuvant (QS21+MPL) only. All vaccine groups have adjuvant (QS21+MPL). Statistical significance was investigated using one-way ANOVA with Tukey’s multiple comparison test and indicate with the mean ± SEM. *; *p* < 0.05, **; *p* < 0.01, ***; *p* < 0.001.

Since the outbreak of COVID-19, the parental SARS-CoV-2 Wuhan strain has continued antigenic changes despite the implementation of COVID-19 vaccination. Here, we investigated differential humoral immune responses in mice after heterologous sequential Wuhan (W), Delta (D), and Omicron (O) spike protein immunizations (W-W-W, W-D-D, W-D-O, W-O-O) or trivalent mixed spike (W+D+O) protein vaccinations, in comparison with the homologous Wuhan spike protein vaccine. Both Wuhan (1 µg) and trivalent 0.33 µg (Tri 0.33: 0.33 µg of each W+D+O BA.1) spike protein vaccination induced a similar level of IgG antibodies specific for Wuhan, Delta, and Omicron variants (BA.1, BA.2, BA.4/5, XBB.1.5) after prime dose (Figure 1). Trivalent 1 µg (Tri 1: 1 µg of each W+D+O BA.1) spike protein vaccination induced IgG to BA.1, BA.2, BA.4/5 at higher levels than those in the W and Tri 0.33 µg prime groups (Figure 1C–F), probably due to a higher dose (3 µg total proteins) of the Tri 1 µg group, which is also similarly observed for IgG to Wuhan and Delta spike receptor-binding domain (RBD) (Supplementary Figure S2A,B).

At 3 weeks after prime dose, heterologous spike first boost dose (W-D, W-O) was IM administered to primed mice (Supplementary Figure S3A–F). All vaccine groups showed highly enhanced levels of IgG antibodies reactive to Wuhan, Delta, and Omicron variant spike proteins (Supplementary Figure S3A–F). The W-D group induced higher levels of IgG to BA.2 and BA.4/5 spike proteins than those in the W-O group (Figure S3D,E). IgG levels reactive to W RBD and D RBD were significantly higher in the Tri 1 µg group than those in the heterologous W-O and homologous W-W groups (Supplementary Figure S2B), also suggesting vaccine dosage effects in Tri 1 µg group.

**Figure 2:**
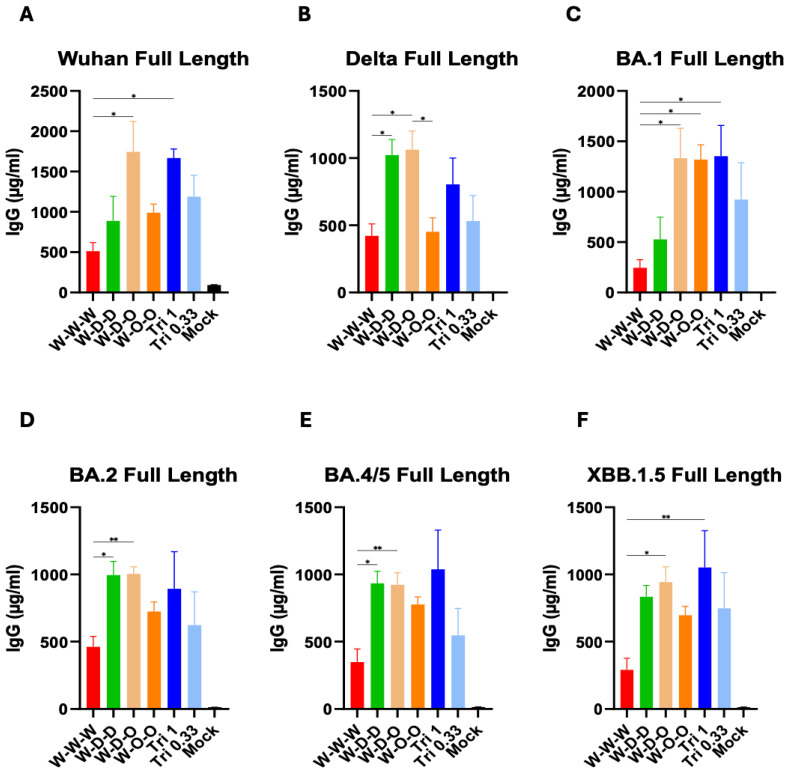
Heterologous or trivalent spike protein vaccinations are more effective in inducing IgG antibodies cross reactive to variants than homologous spike protein repeat vaccination. IgG levels to SARS-CoV-2 variants-specific spike proteins were determined at two weeks after second boost (*n* = 5) by ELISA. (**A**) Wuhan spike, (**B**) Delta spike, (**C**) Omicron BA.1 spike, (**D**) Omicron BA.2 spike, (**E**) Omicron BA.4/5 spike, (**F**) Omicron XBB.1.5 spike. W-W-W: Wuhan (1 µg) homologous prime boost, W-D-D: Wuhan (1 µg), Delta (1 µg) heterologous prime boost, W-D-O: Wuhan (1 µg), Delta (1 µg), and Omicron (1 µg) heterologous prime boost, W-O-O: Wuhan (1 µg), Omicron (1 µg), Tri 1: W (1 µg) + D (1 µg) + O (1 µg), Tri 0.33: W (0.33 µg) + D (0.33 µg) + O (0.33 µg). Mock: adjuvant (QS21+MPL) only. All vaccine groups have adjuvant (QS21+MPL). Statistical significance was investigated using one-way ANOVA with Tukey’s multiple comparison test and indicate with the mean ± SEM. *; *p* < 0.05, **; *p* < 0.01.

A heterologous second boost dose was administered at 8 weeks after the first boost to investigate how IgG antibody responses would be reshaped and further increased. Heterologous sequential spike (W-D-O) vaccination induced significantly higher levels of IgG antibodies reactive to Wuhan, Delta, and Omicron variant spike proteins (BA.1, BA.2, BA.4/5, XBB.1.5) and Delta RBD (Figure S2C) than those by homologous Wuhan spike protein vaccination (Figure 2). Also, the heterologous W-D-D and W-O-O groups effectively induced IgG antibodies to multiple spike variant proteins (Delta, BA.1, BA.2, BA.4/5, XBB.1.5) than the homologous Wuhan group (Figure 2). The Tri 1 µg group showed higher levels of IgG antibodies to Wuhan and Omicron variant (BA.1, XBB.1.5) spike proteins (Figure 2) and RBD (Wuhan, Delta, Omicron BA.1 in Figure S2C) than the homologous Wuhan group, which was similarly observed but less prominently in the Tri 0.33 µg group. There was no significant difference in IgG levels between the Tri 1 µg and 0.33 µg groups after the second boost. These results support that heterologous or trivalent spike protein vaccinations could more effectively induce IgG antibodies cross reactive to variants than Wuhan spike homologous repeat vaccination.

### 3.2. Heterologous Sequential Spike Protein and Trivalent Spike Vaccinations Induce Higher Levels of Receptor Binding Inhibition Activities and SARS-CoV-2 Neutralizing Titers

**Figure 3:**
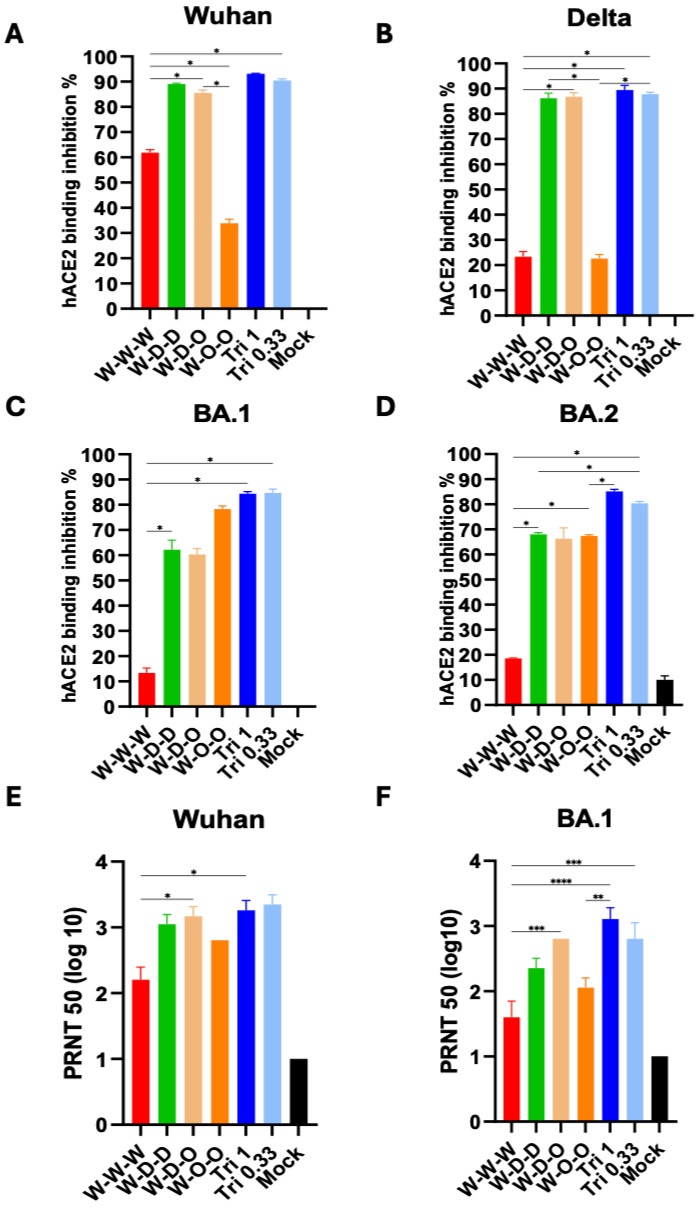
Heterologous sequential spike protein and trivalent mixed spike protein vaccinations induce higher levels of inhibition for receptor binding to variants and SARS-CoV-2 neutralization titers than homologous spike protein vaccination. (**A**–**D**) Inhibition percentage (%) of hACE2 binding to full length spike variants was determined after incubation with 2nd boost sera. Data were showing in 810 dilutions for Wuhan (**A**) and Delta (**B**), 270 dilutions for BA.1 (**C**) and BA.2 (**D**). (**E**,**F**) Neutralizing antibody titers in 2nd boost sera from vaccinated mice (*n* = 5). Vaccine groups are the same as described in Figure 2. Mock: adjuvant (QS21+MPL) only. Statistical significance was investigated using one-way ANOVA with Tukey’s multiple comparison test and indicate with the mean ± SEM. *; *p* < 0.05, **; *p* < 0.01, ***; *p* < 0.001, ****; *p* < 0.0001.

Previous studies reported that receptor hACE2 binding inhibition activities were correlated with SARS-CoV-2 neutralizing titers, a correlate of predictive efficacy [18,19]. Therefore, we determined the relative levels of protective functional antibodies by carrying out an inhibition assay of receptor hACE2 binding to Wuhan, Delta, and Omicron spike proteins (Figure 3A–D). Antisera post-second boost from the sequential heterologous W-D-D and W-D-O groups, as well as Tri 1 µg and Tri 0.33 µg groups, showed significantly higher inhibition activities of hACE2 binding to Wuhan and Delta spike proteins than those from the homologous W-W-W group (Figure 3A,B). The heterologous W-O-O and Tri 1 µg and Tri 0.33 µg groups induced antisera having higher levels of inhibition activities of hACE2 binding to Omicron variant BA.1 spike protein than those from the heterologous W-D-D, W-D-O, and homologous W-W-W groups (Figure 3C). Antisera from the Tri 1 µg and Tri 0.33 µg groups showed higher levels of inhibition activities of hACE2 binding to Omicron variant BA.2 spike protein than those from the heterologous W-D-D, W-D-O, W-O-O, and homologous W-W-W groups (Figure 3D). The receptor binding inhibition activities against Omicron BA.1 and BA.2 variants appeared to be lower than those against Wuhan and Delta spike (810× dilution, Figure 3A,B and 270× dilution, Figure 3C,D). Overall heterologous sequential spike protein and trivalent mixed spike vaccinations were more effective in inducing antibodies inhibiting receptor binding to the SARS-CoV-2 parent and variant spike proteins.

To determine a possible correlation with receptor binding inhibition activities, we performed virus neutralization against SARS-CoV-2 Wuhan and Omicron BA.1 strains by plaque reduction neutralizing titer (PRNT) assay using antisera after the second boost (Figure 3E,F). The groups of Tri 0.33 µg, Tri 1 µg, W-D-O, and W-D-D antisera showed highest titers of PRNT against W strain (2688, 2080, 1972, and 1408 PRNT, respectively, in Figure 3E). The heterologous W-O-O group displayed a moderate level of PRNT (~640), which is higher than the W-W-W (~232) group (Figure 3E). PRNT against O BA.1 strain was observed at higher levels in the groups of Tri 1 µg (~2080), Tri 0.33 µg (~2688), and W-D-O (~1792) than those in the W-D-D (~1408), W-O-O (~640), and W-W-W (~232) groups (Figure 3F). In summary, trivalent mixed spike protein and heterologous sequential spike protein (W-D-O) vaccinations were more effective in inducing neutralizing antibodies against SARS-CoV-2 Wuhan and Omicron BA.1 variant than homologous W-W-W vaccination. Taken together, the results indicate a similar pattern between receptor hACE2 binding inhibition and SARS-CoV-2 neutralizing titers, except for the W-O-O group exhibiting low PRNT titers against Omicron BA.1 variant.

### 3.3. Heterologous Sequential and Trivalent Spike Vaccinations Induce Higher Efficacy of Clearing Lung Virus and Protection against SARS-CoV-2 Than Homologous Spike Repeat Vaccination

**Figure 4:**
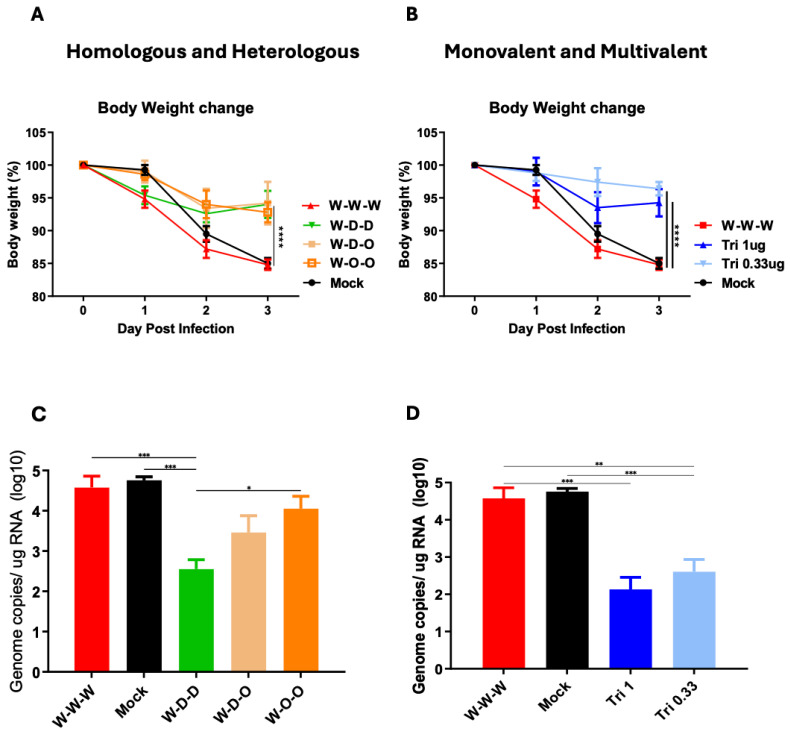
Heterologous sequential spike protein and trivalent mixed spike protein vaccinations induce higher efficacy of protection and clearing lung viral loads after MA10 challenge than homologous spike protein vaccination. Five months after 2nd boost, mice (*n* = 5) were challenged with MA10 (1 × 10^5^ PFU) virus intranasally. (**A**,**B**) Body weight changes were measured for 3 days post-challenge. (**C**,**D**) Lung viral titers (Virus genome copies/ug of RNA in Log10). Vaccine groups are the same as described in Figure 2. Statistical significance was investigated using one-way ANOVA. Error bars indicate the mean ± SEM. *; *p* < 0.05, **; *p* < 0.01, ***; *p* < 0.001, ****; *p* < 0.0001.

Mouse-adapted strain of SARS-CoV-2 strain (MA10) known to be highly lethal in mice [20] was used to test protection efficacy after intranasal challenge (1 × 10^5^ PFU) of vaccinated and mock control mice at 5 months after the second boost (Figure 4A,B). The homologous W-W-W group displayed a severe body weight loss of 15% at day 3 post challenge as similarly observed in the mock control. In contrast, heterologous W-D-D, W-D-O, and W-O-O groups showed moderate levels of weight loss (6–7%) with the W-D-D and W-D-O groups likely recovering at day 3 post challenge (Figure 4A). Consistent with hACE2 binding inhibition and virus neutralization data, significantly less weight loss (3–6%) was monitored in the Tri 0.33 µg and Tri 1 µg groups compared to the W-W-W and mock groups (Figure 4B).

To further support protective efficacy, lung viral loads were determined at day 3 after challenge by quantitative RT-PCR measuring SARS-CoV-2 N gene RNA levels (Figure 4C,D). The heterologous W-D-D and W-D-O groups showed 100- and 20-folds lower lung viral N gene levels, respectively, than those in the W-W-W and mock groups (Figure 4C). More prominently reduced viral RNA levels by 600 and 220 folds were detected in the Tri 1 µg and Tri 0.33 µg groups compared to the W-W-W and mock groups (Figure 4D). These results support that heterologous sequential and trivalent spike vaccinations could be significantly more effective in clearing lung viral loads and inducing protection against SARS-CoV-2 than homologous spike repeat vaccination.

### 3.4. Heterologous Sequential and Trivalent Spike Vaccinations Effectively Prevent Severe Inflammatory Responses Due to MA10 Virus Infection

**Figure 5:**
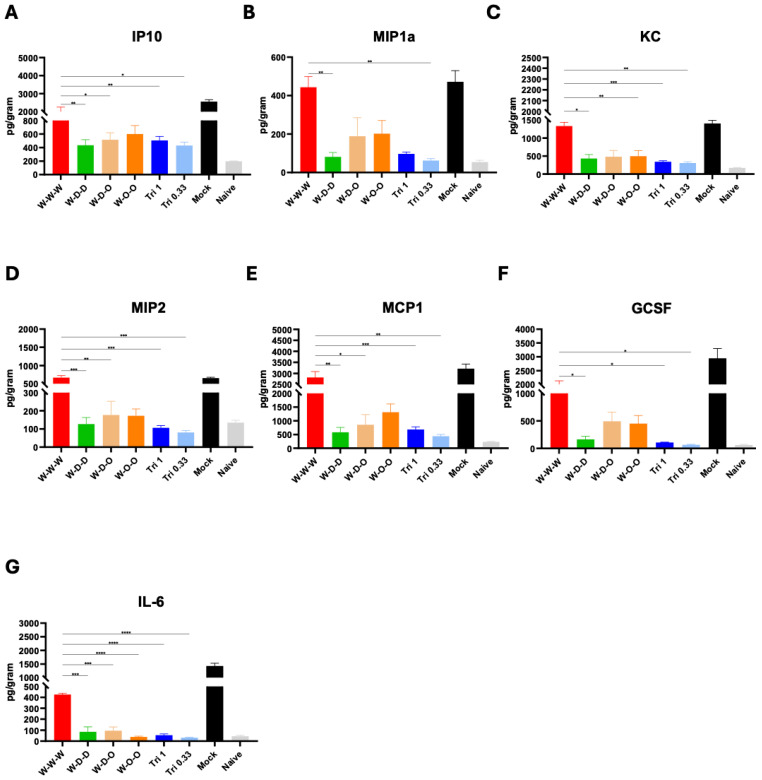
Heterologous sequential spike protein and trivalent mixed spike protein vaccinations effectively suppress inflammatory cytokine and chemokine responses upon MA-10 virus infection. The levels of chemokine IP10 (**A**), MIP1α (**B**), KC (**C**), MIP2 (**D**), MCP1 (**E**) and cytokine GCSF (**F**), IL-6 (**G**) in the lungs of MA10 virus challenged mice were analyzed using Luminex assay. Vaccine groups are the same as described in Figure 2. Mock: adjuvant (QS21+MPL) only after MA10 infection (*n* = 5). Statistical significance was investigated using one-way ANOVA. Error bars indicate the mean ± SEM. *; *p* < 0.05, **; *p* < 0.01, ***; *p* < 0.001, ****; *p* < 0.0001.

SARS-CoV-2 caused severe lung disease by acute induction of inflammatory cytokines and chemokines [21,22,23], which might correlate with weight loss in mice [20]. Therefore, we quantitated inflammatory cytokines and chemokines in lung extracts at day 3 post MA-10 (1 × 10^5^ PFU) virus challenge by Luminex assay (Figure 5). Both W-W-W and mock control groups induced the highest levels of chemokines (IP10, MIP1α, MIP1β, MIP2, KC, MCP1) and cytokines (IL-6, GCSF, IL-1 β) (Figure 5, Supplementary Figure S4), which correlates with rapid weight loss and high viral loads upon MA-10 challenge. In contrast, these inflammatory chemokines and cytokines were detected at the lowest levels in the Tri 1 µg, Tri 0.33 µg, sequential heterologous W-D-D, W-D-O, and W-O-O groups (Figure 5, Supplementary Figure S4), correlating with lower viral loads and less weight loss. These results demonstrate that trivalent spikes and heterologous sequential vaccinations could effectively prevent severe inflammatory responses due to SARS-CoV-2 infection.

## 4. Discussion

With the new emergence of an Omicron variant, the protection efficacy of boost BNT162b2 vaccine was dropped to below 50% and there was low or no efficacy of ChAdOx1nCoV-19 vaccine, even after boosting [9,24]. Breakthrough infections with new variants are commonly observed, resulting in various severity of COVID-19 depending on prior history of vaccination and natural infection, despite the initial high efficacy of COVID-19 vaccines. The initial COVID-19 vaccines based on the original pandemic Wuhan strain had not been updated, despite the evolution to many variants (Alpha, Beta, Gamma, Iota, Kappa, Delta), until the emergence of the Omicron BA.1 variant. Most individuals are presumed to have pre-existing immunity against the original SARS-CoV-2 by either vaccination or natural infections. Therefore, we tested whether trivalent mixed spike or sequential heterologous spike protein vaccinations would be more effective in generating protective humoral immunity, compared to W-W-W repeat vaccination, mimicking early COVID-19 vaccinations in humans. Here we demonstrated that a strategy of multivalent (W+D+O BA.1) or sequential heterologous spike protein vaccinations was significantly more effective in inducing functional antibodies inhibiting receptor binding and broadly neutralizing antibodies against SARS-CoV-2 variants, as well as higher efficacy of protection than homologous spike repeat vaccination.

Trivalent (W+D+O) vaccine was found to be highly effective in inducing cross reactive humoral immunity toward full-length spike Wuhan and Omicron variants (BA.1, BA.4/5, XBB.1.5) beyond the vaccine component, RBD (Wuhan, Delta), and inhibition of receptor binding (W, D, BA.1, BA.2). In line with our findings, previous studies reported that trivalent (W+D+O) S1 subunit or trivalent COVID-19 inactivated vaccine could induce more broadly neutralizing antibodies than monovalent vaccine, but their efficacy of protection was not studied with virus challenge [25]. Tri 0.33 µg dose spike protein vaccine was as effective as Tri 1 µg dose in inducing receptor hACE2 binding inhibition and virus (Wuhan, BA.1) neutralization activities, and conferring protection against MA10 SARS-CoV-2. Receptor hACE2 binding inhibition levels were observed to be correlated with virus neutralization titers in mice [13]. Previous studies reported a correlation between IgG titers of spike antigen binding and receptor hACE2 binding inhibition, as well as between hACE2 binding inhibition and neutralizing antibody titers in human blood samples [26,27,28]. This study in a mouse model suggests that a strategy of multivalent COVID-19 vaccination might be applicable for inducing effective immunity. Quadrivalent seasonal influenza vaccination is safe and highly recommended [29].

Repeated influenza vaccination did not increase the neutralizing antibodies and efficacy of protection against circulating variants and antigenically different strains [14,30,31,32], leading to the induction of immunity toward prior existing epitopes rather than new epitopes present in variants. First boost and second boost Wuhan strain COVID-19 vaccination failed to prevent the emergence of Omicron variants which replaced prior variant Delta [33]. In this study, we tested whether sequential boost with heterologous spike matching variants in Wuhan-primed mice would enhance protective humoral immunity against variants. Omicron boost (W-O) was not as effective as heterologous Delta (W-D) and homologous Wuhan (W-W) in inducing IgG antibodies to Omicron variants (BA.1, BA.2, BA.4/5, XBB.1.5), suggesting less memory B cells with common epitopes between Wuhan and Omicron. After second boost, all sequential heterologous vaccinations (W-D-D, W-D-O, W-O-O) were more effective in inducing IgG antibodies binding to spike variants and neutralizing activities against Wuhan and Omicron strains than homologous repeat W-W-W vaccination. This finding is in line with a study demonstrating that boost with bivalent inactivated vaccine (Beta and Delta) could confer protection against SARS-CoV-2 variants in Rhesus macaques with prior inactivated (CoronaVac) vaccine [34]. Also, boosting with RBD variant-matched adenovirus vaccination was shown to increase neutralizing antibody responses against Omicron subvariants in mice [35]. The results in this study suggest that multivalent strategies (W+D+O) might be more effective in inducing hACE2 receptor-binding inhibition, neutralizing immunity, and clearing lung viral loads than homologous (W-W-W) or heterologous sequential vaccinations.

Unexpectedly homologous (W-W-W) spike protein vaccination induced low levels of inhibition activities against receptor binding to Wuhan spike and low neutralizing titers against Wuhan strain, which is correlating with no protection against MA10 virus challenge. MA10 virus was selected after multiple passages in mouse lung tissue, acquiring the capacity to replicate at high lung viral titers. During adaptation to high affinity mouse ACE2 binding, the MA10 virus acquired mutations in the spike protein compared with Wuhan strain [20,36]. These mutations are also found in the RBD of Alpha (B.1.1.7) and Beta (B.1.351) variant. Homologous spike vaccination might limit the breadth and flexibility of immunity, leading to low efficacy of protection against MA10 with several mutations. Adjuvanted monomeric RBD protein vaccination induced low neutralizing titers and low efficacy protection against MA10 as high lung viral loads and weight loss in mice [37]. Hamsters with monomeric full length spike protein vaccination exhibited high lung viral loads and severe disease [38]. Taken together, a strategy of multivalent spike antigens with updated strains predicted to circulate dominantly in near future would be superior to the monovalent repeated vaccination, reflecting annually updated quadrivalent influenza vaccines.

In conclusion, this study demonstrated that multivalent or sequential heterologous spike protein vaccinations was significantly more effective in generating protective antibodies inhibiting hACE2 binding and cross neutralizing antibodies against SARS-CoV-2 variants, and in conferring higher efficacy of protection than homologous spike repeat vaccination. Future studies will need testing virus neutralization activities and protection against currently circulating SARS-CoV-2 variants. It is also important to investigate vaccination strategies to further enhance recall-immune responses to the first vaccination or first exposure to pathogens using an animal model and multivalent and sequential heterologous spike encoding mRNA vaccine platforms.

## Data Availability

Data are contained within the article.

## Supplementary Materials

The following supporting information can be downloaded at: https://www.mdpi.com/article/10.3390/vaccines12040362/s1, Figure S1. Human ACE2 receptor-binding levels of SARS-CoV-2 full length spike ectodomain recombinant proteins. Figure S2. IgG antibody responses against variants specific RBD in BALB/c mice two weeks after each immunization. Figure S3. Variants-specific IgG antibody responses in 1st boost immune sera. Figure S4. Cytokine and chemokine levels in the lungs of MA-10 virus challenged mice.
