# Supplementary material for: Multivalent and Sequential Heterologous Spike Protein Vaccinations Effectively Induce Protective Humoral Immunity against SARS-CoV-2 Variants
Source: Vaccines (Basel). 2024 Mar 27;12(4):362. doi: 10.3390/vaccines12040362 (PMC11053539; doi:10.3390/vaccines12040362)
Supplement: Supplementary file 1 [file vaccines-12-00362-s001.zip › vaccines-2926803-supplementary.pdf]

Multivalent and sequential heterologous spike protein vaccinations effectively induce protective humoral immunity against SARS-CoV-2 variants

Rong Liu [#1], Janhavi P. Natekar [#2], Ki-Hye Kim 1, Heather Pathak [2], Noopur Bhatnagar [1], Jannatul Ruhan Raha [1], Bo Ryoung Park [1], Anchala Guglani [2], Chong Hyun Shin [1], Mukesh Kumar [*,2] and Sang-Moo Kang [*,1]

1 Center for Inflammation, Immunity & Infection, Institute for Biomedical Sciences, Georgia State University, Atlanta, GA 30303, USA.

2 Department of Biology, College of Arts and Sciences, Georgia State University, Atlanta, GA 30303, USA

*Correspondence: author: Sang-Moo Kang <skang24@gsu.edu>Mukesh Kumar <mkumar8@gsu.edu>

#Equally contributed to this study

Supplementary Fig S1.

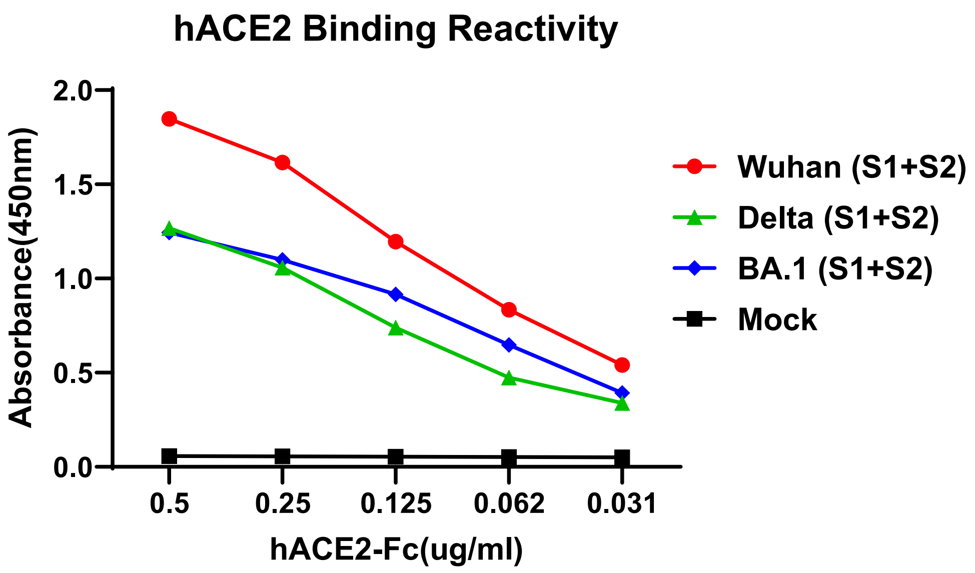

Figure S1. Human ACE2 receptor binding levels of SARS-CoV-2 full length Spike (S) ectodomain recombinant proteins. The receptor binding reactivity of the protein used for immunization was investigated using serially diluted hACE2-Fc (0.5 – 0.031) on the 96-well plates precoated with spike proteins at 80ng/100ul.

Supplementary Fig S2.

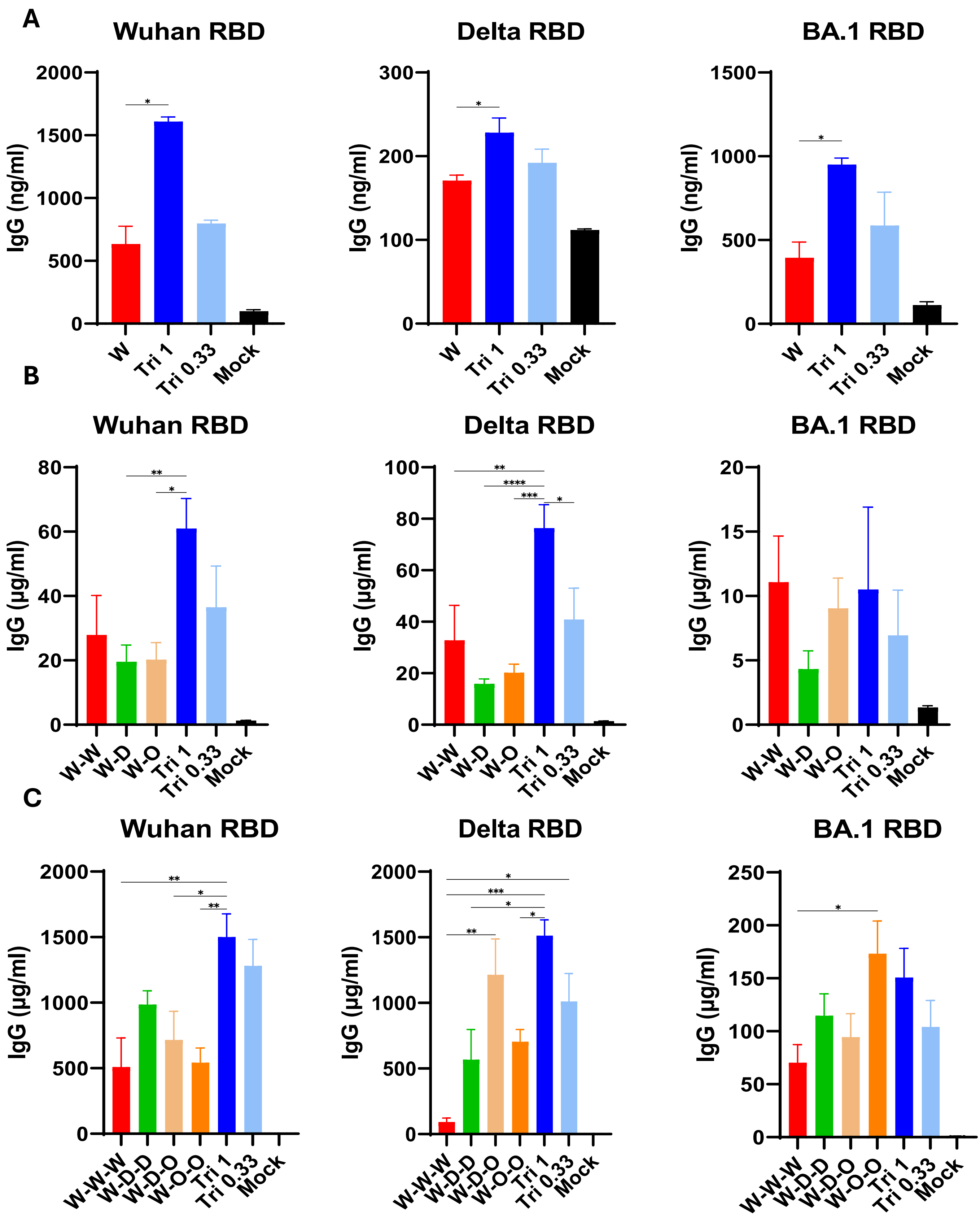

Figure S2. IgG antibody responses against variants specific RBD in BALB/c mice two weeks after each immunization. IgG to variants specific RBD in prime and boost sera. (A) IgG response to Wuhan, Delta, and BA.1 RBD in two weeks after prime sera. (B) IgG response to Wuhan, Delta, and BA.1 RBD in two weeks after 1st boost sera. (C) IgG response to variants RBD in two weeks after 2nd boost sera. Statistical significance was investigated using one-way ANOVA. Error bars indicate the mean ± SEM. *; $p < 0.05$, **; $p < 0.01$, ***; $p < 0.001$, ****; $p < 0.0001$.

Supplementary Fig S3.

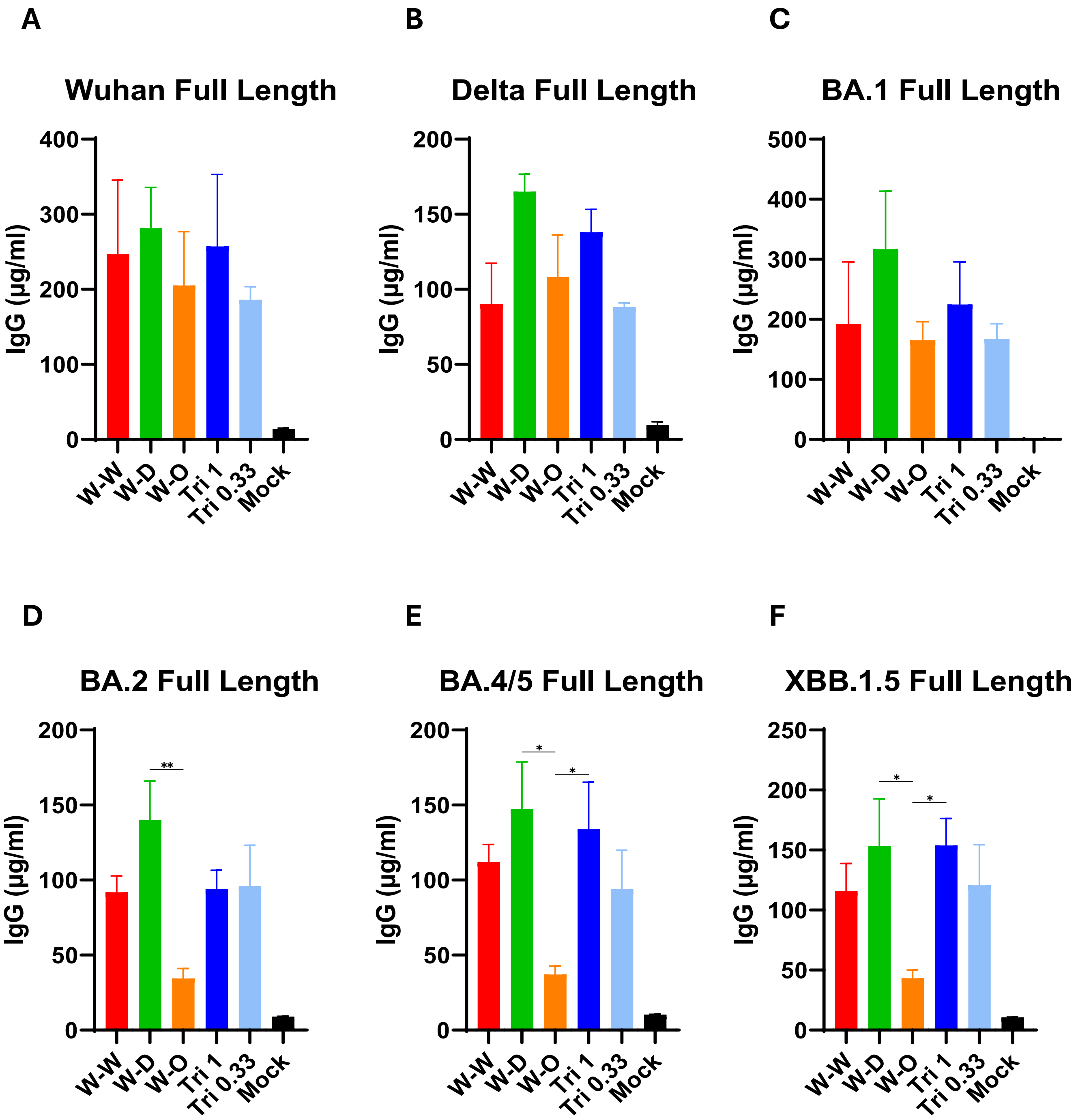

Figure S3. Variants-specific IgG antibody responses in 1st boost immune sera. BALB/c mice (n=5) were intramuscularly (IM) boosted with Wuhan, Delta, Omicron, or trivalent vaccine. Variants-specific antibody responses were analyzed by using ELISA (A-F) IgG specific for different SARS-CoV-2 variants full-length spike in immune sera were collected 2 weeks after boost. Statistical significance was investigated using one-way ANOVA with Tukey's multiple comparison test and indicate with the mean ± SEM. *; $p < 0.05$, **; $p < 0.01$, ***; $p < 0.001$.

Supplementary FigS4.

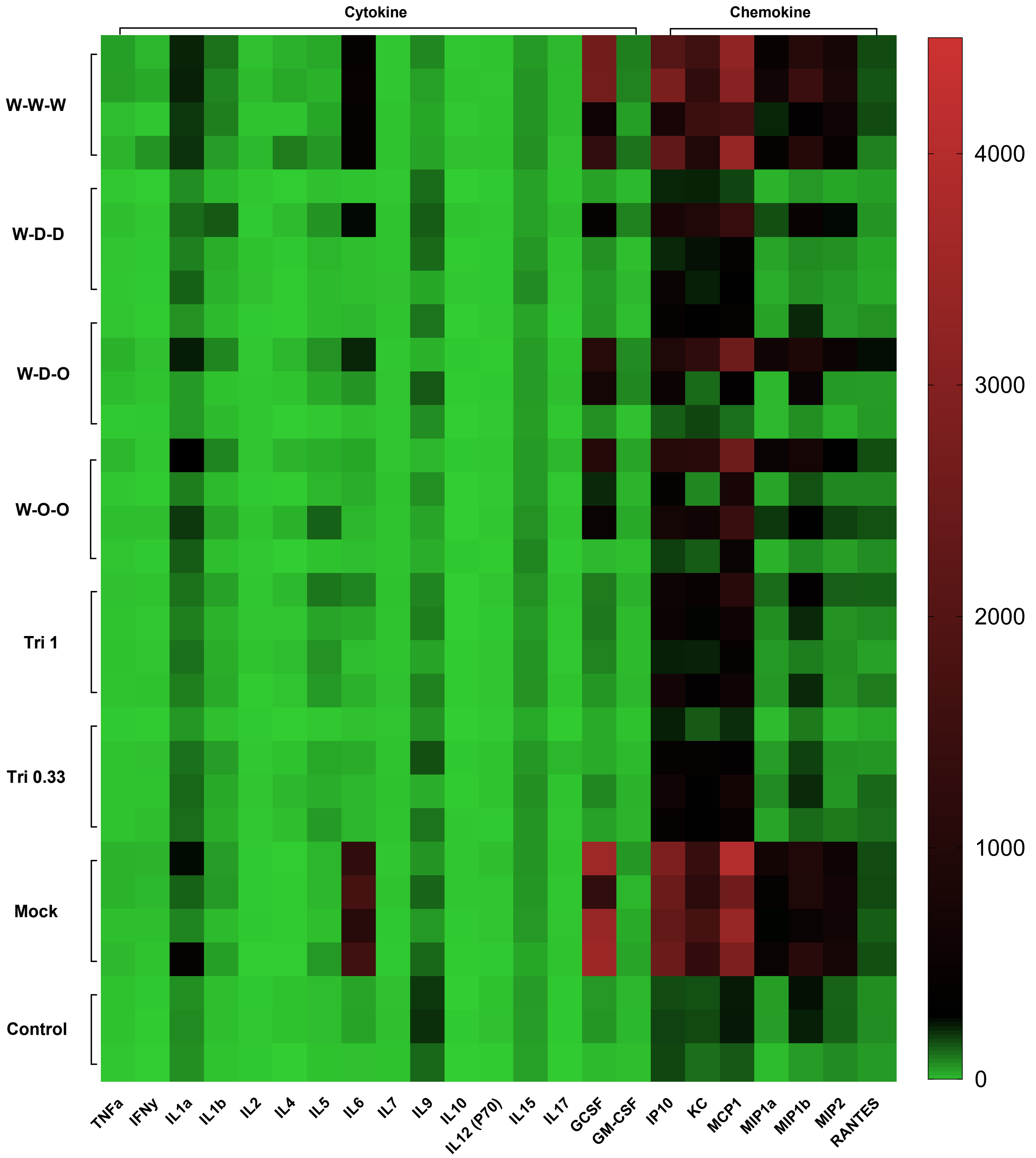

Figure S4. Cytokine and chemokine levels in the lungs of MA-10 virus challenged mice. Cytokine and chemokine profiling of immunized mice after challenged with MA10 virus (n=4-5). Heatmap analysis was conducted using GraphPad Prism 9.5.1.733 software package.
